# Editorial Chit-Chat

**Published:** 1876-04

**Authors:** 


### Editorial Chit-Chat.

OUR CORRESPONDENCE.—Read it. You will find an unusual number of interesting and racy letters.

IS IT YOU?—Some of our subscribers have not yet sent in their subscriptions for this year. Please see that you are paid up at once, as we dislike to cut off any of our readers.

RETURNED.—H. has returned from Florida, but not in season to give us his experience among the alligators and other aquatic game of that clime. Perhaps he will favor us in our next.

WHERE, O, WHERE, is the "Elmira Medical and Surgical Association?" Has the association and his "associate" departed for ever, to leave a suffering community to struggle unaided and uncured?

IN ARREARS.—Very many of our subscribers have failed to send in their subscriptions for this year. We hope they will do so immediately, the sum is small, so that the prevailing excuse of "hard times" cannot hold good in this instance. Send in your 55 cts.!

POOR ROBIN.—This morning we saw a robin—an early bird, standing in the snow, up to his belly, sadly singing "spring time has come." We thought he was a little "out of sorts"—probably had sore throat, for his song was miserably out of tune, but strangely in keeping with the weather. We never before saw a robin searching under the snow for worms. None but anglers—bate fishermen, you know—would think of such an experiment.

HARTZ.—There is no species of entertainment to which we are so fascinatingly attracted, as to those given by magicians, and no one is more capable of affording this amusement than Prof. Hartz, who recently gave a week's entertainment of this character in our city, and who is now exhibiting in the eastern states. We are taught to believe that our eyes are less capable of being deceived than any of our organs of sense, but Prof. Hartz will speedily convince us that our optics, even, cannot be relied upon. His entertainment is the very best of the kind, and the Professor himself a gentleman, whom it is a pleasure to know. We recommend our eastern subscribers to see Hartz by all means.

FLORAL DECORATIONS.—Park Church, in this city, has become noted for the floral displays about its pulpit. The lady who takes great pleasure in the arrangement of the flowers, exhibits most exquisite taste, and affords much pleasure to, while her flowers elicit the admiration of all who attend upon worship in this edifice. The congregation have learned to look for the floral offerings, as regularly as they do for the pastor himself.

---

TRICKS OF THE TRADE.—Coming through Wisner Park a few mornings since; we overheard the following conversation between two workmen, who, with lunch pails in hand, were hastening to their daily labor: "Jim, how long can you make that job *last*, up yonder?" "O, guess we can squeeze two more weeks out of it yet," was the reply. We eyed those two particular carpenters quite attentively, and concluded they would never be employed upon any "job" of ours.

---

WISE DOCTORS, THEY!—We see from the newspaper reports of the Penn Yan murder case, that the coroner, with two physicians, disinterred a body, thought to have been murdered with poison; that they removed from it what they supposed to be the stomach, but which upon inspection, proved to be the *heart and lungs* of the subject. The story seems incredible, but if true, those particular doctors are in sad need of further instruction in anatomy, their former attainments and efforts in that direction seemingly having amounted to *nil*.

---

BUSY AND DIRTY.—We had occasion to visit Scranton, Pa., a few days since, and found it a very busy and exceedingly dirty place. Perhaps the one quality is inseparable from the other, by reason of the character of their work, engaged as they are, in mining and in the extensive manufacture of iron and steel. But we could see no excuse for the inhabitants emptying their swill into the gutters, or out at their back doors, upon the surface of the ground. If the city authorities keep a mortality record, we should like to see how many deaths are reported the coming summer, under the head of "typhoid fever."

---

RELIABLE MEDICINES.—It is a great desideratum with the physician to have his medicines in a compact and agreeable form for administration. Many of our most potent remedies are so abominably nauseous that they become difficult, and in many instances, impossible of administration. Wm. R. Warner & Co., the well known manufacturing chemists of Philadelphia, have come to the relief of the physicians in this respect, and have prepared pills from all the drugs that can be given in this form, and have sugar coated them, so that they can be administered without bringing the drug in contact with the mouth. The favorite formulæ, of celebrated physicians too, are prepared in this manner, containing in every instance, *pure* medicines, so that physicians have learned to rely upon these preparations. Any physician can send his prescriptions to these chemists and have pills or elixirs prepared, of undoubted purity, and so secure a safe, reliable and agreeable means of administering medicines. We have tried them, and know whereof we speak.

---

"HOW TO ACQUIRE AND PRESERVE HEALTH," is the title of a large and handsomely bound volume, published by H. N. McKinney & Co., 725 Sansom street, Philadelphia. The book consists of thirty-eight popular lectures, as given by Dr. C. W. Gleason, the author and lecturer, several years ago, and who is so well and favorably known throughout the country. The book abounds in good hints upon matters of health and disease, and has also a valuable appendix containing antidotes for poisons, and other useful formulæ.

---

TROUTING.—Now is the season to get your rods in order for the summer campaign after trout. Considerable pleasure can be gleaned from mending a broken tip, varnishing your rods, oiling and cleaning your reels, and manufacturing a stock of favorite flies; for, of course, you are a fly fisherman and would not descend to the ways of the pot hunter, by taking the gamey trout by any other means than with the fly.

Before another BISTOURY reaches our readers, we hope to have had our usual vacation in the woods, when, perhaps, they may hear of our success and pleasure.

---

A RIDE ON A LOCOMOTIVE.—Taking a trip down the Lehigh Valley Railway recently, we were invited by Superintendent Robert A. Packer, to ride upon one of their elegant locomotives, Mr. Packer acting as engineer. He seemed to be perfectly familiar with all the machinery of the engine, and ran and stopped it with the precision of an experienced engine driver. Perceiving the mile posts flying by us somewhat astonishingly fast, we took our watch in hand and timed their flight, when we discovered that but one minute and eight seconds of time was consumed in running one mile, or at the rate of fifty-three miles an hour. Notwithstanding our rapid run, no unpleasant lateral motion was communicated to the engine, nor the startling, jumping and bounding, that we have experienced on other roads. The Lehigh Valley track is in most excellent condition, steel railed, well equipped throughout, and they *invariably* bring their trains in on time.

This is not a puff—we paid our fare.

---

THE ELMIRA WATER CURE.—This very pleasant and popular home for invalids has been filled with those in search of health and rest, during the entire winter months. The Institution, in addition to its many natural attractions, has a strong medical faculty, in the persons of S. O. Gleason, M. D., Rachel B. Gleason, M. D., Adele A. Gleason, M. D., Theron A. Wales, M. D., and Zippie Brooks Wales, M. D. The Cure will be in readiness to receive their summer guests and patients by the first of May. No better place exists anywhere, and no more capable physicians for the treatment of chronic ailments, than at this institution.

---

FLORIDA.—Many of our friends are just now enjoying the pleasant weather, delightful scenes, and luscious fruit of the land of flowers. The next best thing to do, when unable to go to Florida yourself, or even if you *are* going, is to purchase a copy of CAMP LIFE IN FLORIDA, published by the Forest and Stream Publishing Co., New York. It is a capital book, brim full of just such information as would be appreciated by the sportsman tourist, in Florida. It tells you where to go to find the best game and fish, what to provide yourself with, where to stop and who to secure for guides or boatmen, besides an incredible amount of information about points and places in this truly beautiful land.

---

THAT ROYAL TRAIN.—On the second day of February last, a lady of this city, left Indianapolis, by the "Bee Line" to Cleveland, destined to her home. She purchased her ticket through, by the so-called "Royal Train," which, through the inexcusable negligence of the conductor, on that particular train, proved to be a royal disappointment, of the most vexatious character.

Just before entering Cleveland, the conductor gathered his tickets, as usual, examined the lady's through ticket, and informed her she would have twenty minutes for breakfast, at the Cleavland depot, and deliberately carried her by the Great Western junction, where he should have left her, and where the train was in waiting to carry her home. By this piece of carelessness, the lady was left from 7 o'clock in the morning, until 9:30 at night, before she could take another train home.

One lady, since, has been left in precisely the same manner, and one gentleman, also, who are loud in their denunciation of that "Royal Train." Had not the managers of the "Bee Line," from Indianapolis to Cleavland, better secure conductors who will pay more attention to the comfort and convenience of their passengers, and direct them to inform the traveler that the Atlantic & Great Western junction is one mile west of Cleveland, and that to go to the Cleveland depot, insures their imprisonment in that city, for fifteen hours? We think so, else may some justly indignant person bring in a claim against the Company for "false imprisonment," or something of that sort.

---

COSMOLINE.—We have seen this preparation advertised in the medical journals of the land, and had concluded it to be some "patent medicine,", on a par with the many nostrums that are offered for sale, until our attention was called to its great adaptability to the manufacture of salves, and as a vehicle in which to incorporate various medicines for local applications. We have tried it, and find that cosmoline is the very article so long desired by physicians with which to prepare their unguents, and is a very agreeable and effective agent applied to chapped or chafed skin of infants or adults. It is so bland, free from acids or other irritating materials, and does not become rancid by age, that it becomes the very best means through which to introduce medicines into the eye, in cases where simple cerate or starch emulsion is generally employed. We are delighted with cosmoline and recommend it to the profession in all cases where simple cerates and other stale unguents have heretofore been employed.

---

MINATURE MINE.—We hear of a minature mine manufactured by a couple of mechanics of Scranton, a description of which seems somewhat astonishing. We are informed that it represents a section of a coal mine, with miners at work, picking, drilling and blasting; cars drawn by mules, and attended by boys, are conveying the mined coal to the shaft, where it is raised to the valley above ground, and then conveyed to the breaker, where it is crushed, screened, and sorted. The usual boys are seen picking the slate from the coal, while the overseer stands over them with his long whip, to keep them from play, and to direct their eyes upon their work. The coal is dumped after being sorted, into the train of cars below, where the railroad is seen, with the long line of coal dumps, drawn by the puffing, snorting engine. Upon the ground is seen the blacksmith shop, with men at work sharpening picks and drills, "and they *do* work," our informant said, as naturally as though alive. In short, all the machinery, and all the different processes which coal goes through, until prepared for the market, is represented faithfully, and with wonderful accuracy, in this automatic mine. The machinery is driven by two eight horse-power engines, made by the same workmen who constructed the model. The entire device occupies a space six feet long, by three or four wide, the men at work being about four inches tall. These men, we neglected to say, are dressed precisely as the miners, even to the little lamp, which is seen burning upon their caps, while all their movements are in exact imitation of the miners at work.

It is the intention of the skillful makers to exhibit their mine at the Centennial, of course, and we are sure it will attract much attention, even among the multiplicity of other curious things that will be there to be seen.

---

CENTENNIAL TIMES.—These be times of rejoicing for our prosperity and national existence. In view of recent developements, it is very meet and proper that we should rejoice in the possession of a government and a country at all. Is it not humiliating that those high in authority, men who should be above a breath of suspicion, have been discovered in the perpetration of crimes and misdemeanors which we are accustomed to see attributed to the very lowest and most depraved members of community? When cabinet officers, executive attaches, and other prominent and noteworthy men of the nation, descend to the practices of wily and corrupt politicians, in truth, we should celebrate the hundredth anniversary, if for no other reason than for thankfulness that we have been able to exist so long as a nation, under such miserable and corruptible leadership.

It requires no political economist to foresee a disastrous termination to our national existence, long before another centennial is reached, unless the *people* awake to the exigencies of the case, by seeking to have honest and capable men represent them in the primary as well as the more important elections of the state and nation. Too much countenance is given to the practices of professional politicians, who, in the main, are as dishonest as they are successful in managing party machinery. We vote too much for *party* and too seldom for honest, capable men. There is not enough "bolting" from party nominations, but a too willing acquiescence in the selection of men for office, who are unworthy of our respect and support. We are not wise enough to point out a remedy for this evil of voting for men who buy, or otherwise intrigue for their nominations, and who are wholly unsuited for the positions sought, but we can refuse to vote for them, and can cast a ballot for a candidate of our own nomination, whom we do believe to be both honest and capable.

Perhaps, if all men, who think as we do, and they must be numerous, were to get together in every city and village, and nominate independent, honest, upright men for office, and then vote for them, a step will at least, have been taken in the right direction. From all this, it is plain to be seen, that we are a very poor politician, and it is equally apparent that we are supremely disgusted with the present management of our national affairs.

---

PIGEON SHOOTING.—The woods have been "alive" with wild pigeons in Bradford County, Pa., during the past month. Encouraged by the representation of friends, who had been upon the ground, and had carried home immense numbers of the feathered tribe, we spent one day on the mountain top, where the flights were said to be the greatest. Having traveled forty miles by rail, and secured comfortable quarters at a friendly farmer's house—the hotel of the village being crowded with nimrods—we climbed the steep mountain, at early dawn the next morning, and with a friend, sat down in a "clearing" to await the coming of the birds. And soon they *did* come—millions of them, and from every conceivable direction, in swarms, acres in extent, until the sun was obscured by their flights. After recovering from our surprise and awakening to the realization of the fact that we were in possession of an excellent breech-loader, and one hundred and fifty cartridges, we, that is, with our friend, commenced firing—first by sections, then by platoons, until the hills rung with the echoes of our light artillery. And just so did about one hundred other sportsmen, who had arrayed themselves in elegant skirmish line along the brow of the hill, converting the territory into a most excellent representation of a battle field. And you should have seen those poor bird's bewilderment—take whatever direction they chose, bang, bang, bang, was their greeting, from every nook and corner of that hillside, causing them to wheel, turn, countermarch, oblique, and double-quick to every point of the compass, and then settle on the tree tops seemingly to determine what in fury was up, and what was best to be done under the circumstances. This meditation, on their part, was fatal to many of them, and reduced them to many a potpie, and their survivors to a complete state of anarchy and confusion, so that by evening, they abandoned the territory, and for aught we know, the state of Pennsylvania. Reader, go pigeon shooting the very first opportunity! It is an experience the remembrance of which will remain with you for a lifetime.

---

THE POCKET GYMNASIUM.—We have it,—"got it bad," as the slang phrase expresses it, and are upon the verge of despair, not knowing whether to proclaim it a "blessing in disguise," or an abomination in bold relief. Calling at a friend's house, he invited us to feel of his muscle, declared he "developed" his chest from two to four inches —more or less,—had made the muscles of his arms and chest as hard as iron, and was then engaged upon the certain process of "developing" his legs, although we could see no real necessity for any effort in that direction. Well, enticed and persuaded into the purchase of the "pocket gymnasium" by our enthusiastic friend, we immediately went into training. Arose promptly at seven o'clock, upon being notified so to do by the ringing of the telegraph bell from the Institute, by another chap who was up and "in training" too, at the other end of the line, and went to work. First we pulled the "number six," twenty times, while standing in our night gown, with the thermometer standing likewise, at two below zero. We then increased the rapidity of the movement, to accomplish the same thing with our circulation, and to prevent the blood freezing in our veins. By the time we had stretched that confounded rubber thing twenty more times, our head was in a glow and our feet at the freezing point, while we, ourself,—i. e. what was left of us, couldn't lift a feather or heave a sigh. So we went to bed and warmed our icy feet against our better-half, and then arose valiantly to try it again. We took twenty more pulls at it, trying to get ourself in every position which is so graphically displayed upon the illustrated circular, while our spouse watched us from beneath the comfortable rose blankets and inquired whether we were practising for a pantomime or lunatic asylum. We paid no attention to this cruel observation, but diligently persevered in the stretching of that gumelastic tube, until our flexors and extensors utterly refused to engage in that healthful but exceedingly laborious exercise longer, when we arranged our toilet and departed upon our morning walk. All went well enough "until the even had come," when our arms felt as though they had been flailed right diligently by a farmer laddie in full possession of all his muscular vigor. With a pillow upon the seat, and another to our back, we ensconsed ourself in the big, easy chair, and was fed our oat meal and cream by other and stronger hands than ours. While occupying this comfortable position, and arguing with the chap who runs the gymnasium at the other end of the line, that we had *not*—why, of course not—exercised too violently at the start, behold the younger members of the household were seized with the mania for gymnastic muscular development, and the way that "pocket gymnasium" was handled by these four urchins, would have done credit to the most renowned acrobatic troupe. We have not the time, space nor patience to describe the wonderful results of that brief hour of family exercise, nor to record how our youngest fell from the stool (in trying to imitate that chap in the picture, with a pen behind his ear), and bumped his head, until a smaller one was developed, the size of an orange; nor yet how one of the "stretchers" slipped and gave baby a black eye; nor how the mania spread to the kitchen girls, who were found practising with the flat irons and our best Sunday suspenders. All this seems like a dream from which we have happily awakened, to find our pocket gymnasium hidden in the further end of our closet, awaiting the time when our arms shall be reported able for duty. Procure a "pocket gymnasium" by all means—"it's a big thing!"

---

RECEIPTS.—Many of our subscribers ask us to send them receipts for subscriptions paid. This we cannot undertake to do in a circulation as large as ours. One clerk would have to be employed for that purpose alone, an expense that cannot be incurred in a low priced magazine like ours. On the second page of the cover we undertake to credit all subscriptions received up to the time of going to press, usually as late as the 25th of the month. If your name does not appear, write us, and we will undertake to discover where the fault lies. If you desire an answer, do not forget to enclose a postage stamp, else you will receive no reply. Please remember also, that we cannot send "sample copies" of journals with which we club. For these, apply to the publishers. If your journal fails to come to hand after a reasonable time, write to the publishers; if they do not reply, *then* write us, enclosing stamp, and we will rectify the difficulty. Our subscribers will spare us much unnecessary trouble by heeding what we have here written.

